# Underutilization of Research Journals by Undergraduate Students of Medical Colleges in Islamabad: A Cross-sectional Study

**DOI:** 10.7759/cureus.2568

**Published:** 2018-05-02

**Authors:** Fahad Azam, Abida Shaheen, Fuad Shaikh, Abdul Ahad E Sheikh, Fasih Sami Siddiqui, Anoosh Zafar, Nismat Javed

**Affiliations:** 1 Department of Pharmacology and Therapeutics, Shifa College Of Medicine, Islamabad, PAK; 2 Department of Pharmacology & Therapeutics, Islam Medical College, Islamabad, PAK; 3 Student, Shifa College Of Medicine, Islamabad, PAK; 4 Student, Rawal Institute of Health Sciences, Islamabad, PAK; 5 Shifa College Of Medicine, Shifa Tameer-E-Millat University Shifa International Hospital, Islamabad, PAK

**Keywords:** restricted access, research articles, medical curriculum, assessment

## Abstract

Objective: To analyze the factors behind the underutilization of research articles as an adjuvant source of knowledge by medical students.

Materials & Methods: We conducted a cross-sectional study of students from medical colleges in Islamabad from June 2017 to August 2017. The students were verbally informed about the study, and those who gave their consent were included. The data was collected through a self-constructed questionnaire. Cronbach's alpha was used to assess the internal consistency of the questionnaire, and it was found to be 0.68. The data obtained was analyzed on IBM's statistical package for the social sciences (SPSS) version 21 (IBM, Armonk, NY, US).

Results: A total of 382 students participated in the study. The use of research articles for the preparation of problem-based learning (PBL), small group discussions, or assessments was very low. Students did, however, consult journals if emphasized by the faculty. A majority of the students did appreciate the importance of medical journals to explore detailed information about disease states and health issues encountered by self or family members. The use of research articles by students for preparing for exams was very low.

Conclusion: The students’ underutilization of journals may be attributable to an over-familiarity with books, a lack of faculty prompting, and a lack of knowledge on how to access such journals. These factors should be addressed while designing the medical curriculum to enhance journal perusal among medical students.

## Introduction

Research is of great consequence within medicine because of the effect it has on the evidence-based medicine (EBM) practice of healthcare professionals and the training of medical students [1-3].The latest research can be accessed via numerous online medical literature databases serving as an excellent alternative source of learning because they provide the most up-to-date information backed with statistical evidence [4]. In a study that established the Internet as one of the modes of publicity for research, 206 (68.7%) students had already participated in research as a principal researcher, co-researcher, or research volunteer [5]. Taking into account the modern trends in medical education that aim at making students lifelong and self-directed learners, keeping oneself abreast with the latest updates in the field of medical sciences becomes more significant [6]. However, it has been observed that a majority of medical students do not utilize research journals to enhance their learning due to multiple factors. The aim of this study is to investigate the reasons and various obstacles behind the perceived low usage of research journals as a study tool among undergraduate medical students.

## Materials and methods

We conducted a cross-sectional study of students from medical colleges of Islamabad from June 2017 to August 2017. The students were verbally informed about the study, and those who gave their consent were included in the study.The data was collected through a self-constructed questionnaire in collaboration with medical education experts. The idea of the questionnaire was conceived by the teaching faculty members, keeping in mind the students' feedback of various modules. Cronbach's alpha was used to assess the internal consistency of the questionnaire, and it was found to be 0.68.

The questionnaire assessed the frequency of use of research journals as a source of learning as well as the benefits of research journals by using a five-point Likert scale (Never, Less Often, Often, More Often, Always) to quantify responses for the statements provided. It was also used to record means of access to research journals using a three-point Likert scale (Never, Sometimes, Always) to quantify responses. The participants of the study also had to choose from a number of statements about the various barriers that are faced in the research journal-oriented learning process.

The data obtained was analyzed on IBM's statistical package for the social sciences (SPSS) version 21 (IBM, Armonk, NY, US). Descriptive statistics were used for the analysis and description of the data. Frequencies and percentages were calculated for participant distribution according to the academic year, utilization of journals, and means of access to the journals. The independent sample t-test was used to find the association of underutilization with various variables.

## Results

**Figure 1:**
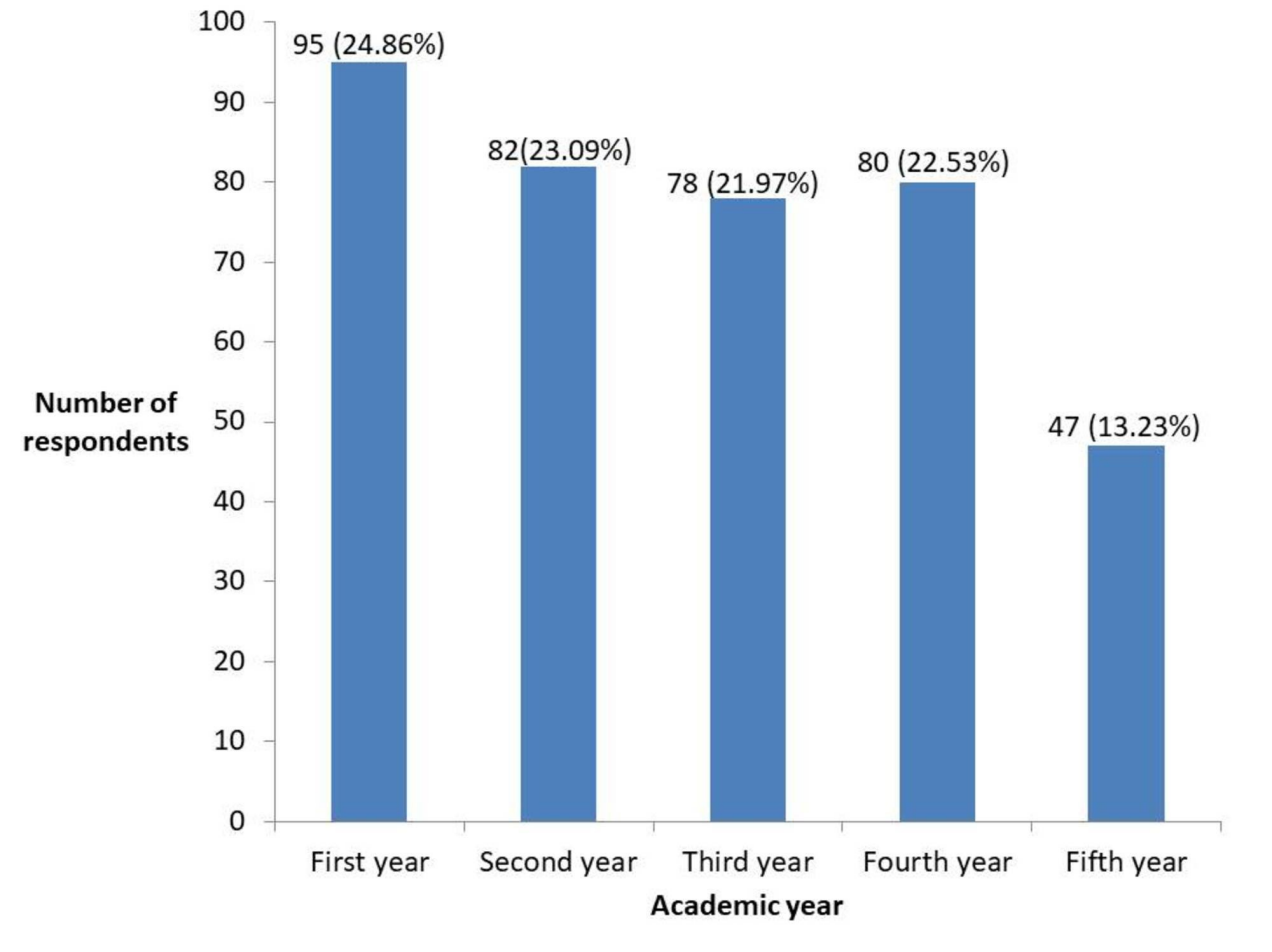
Distribution of the participants according to their academic year

A total of 382 students responded to the questionnaire (Figure 1).

Based on the participants' choice from among the various statements of the questionnaire, it was evident that only six participants (1.6%) used journals as resource material along with their textbooks. The independent t-test was applied to assess if the differences in the number of responses obtained were significant. A p-value < 0.05 was considered significant (Table 1).

**Table 1:** Utilization of medical journals for different purposes by medical students p-value < 0.05 was considered significant

Statements	Responses					
	Never	Less Often	Often	More Often	Always	p-value
Use of medical journals as an adjuvant source of learning to books	240	112	19	5	6	0.00
As a sole source of learning about a particular topic	281	85	9	4	3	0.38
Promptness by faculty to consult journal articles	145	132	73	21	11	0.00
To prepare for problem-based learning (PBL)	226	96	39	10	11	0.07
To prepare for small group discussion sessions	290	79	7	1	5	0.01
To prepare for exams	305	61	12	2	2	0.20
To prepare for clinical rotations	323	29	18	9	3	0.00
For personal or family health issues	191	105	64	16	6	0.34

The questionnaire also prompted the participants to choose from a list of means of access through which they utilized the research journals. It was found that 167 participants (43.7%) always used the Internet to access journals. The independent t-test was applied to assess if the differences in the number of responses obtained were significant. A p-value < 0.05 was considered significant. This has been presented in (Table 2).

**Table 2:** Means of access to the utilization of medical journals p-value <0.05 was considered significant

Statements	Responses			
	Never	Sometimes	Always	p-value
Use of the Internet to access research articles	39	176	167	<0.01
Use of library to find research articles	255	110	17	0.00
Use of newspapers to access research articles	268	100	14	0.12
Consulting faculty for the provision of journal articles	304	68	10	0.00

A majority of the students did not agree upon the utilization of journals as the only tool of learning and most of them never consulted online research articles for preparing for problem-based learning (PBL), small group discussions, or assessments. However, a mixed response was observed regarding the emphasis of faculty members on pursuing journals for the learning need of students. A total of 43% students highlighted the need for the Internet facility to search for relevant online scientific information whereas 67% students did not consult the library to find research articles. Four percent of students perceived only newspaper to be an adequate source for any kind of latest scientific information while 3% students approached the faculty for the provision of medical journals. However, most of the students did appreciate the importance of medical journals to explore detailed information about disease states and health issues encountered by self or family members.

**Figure 2:**
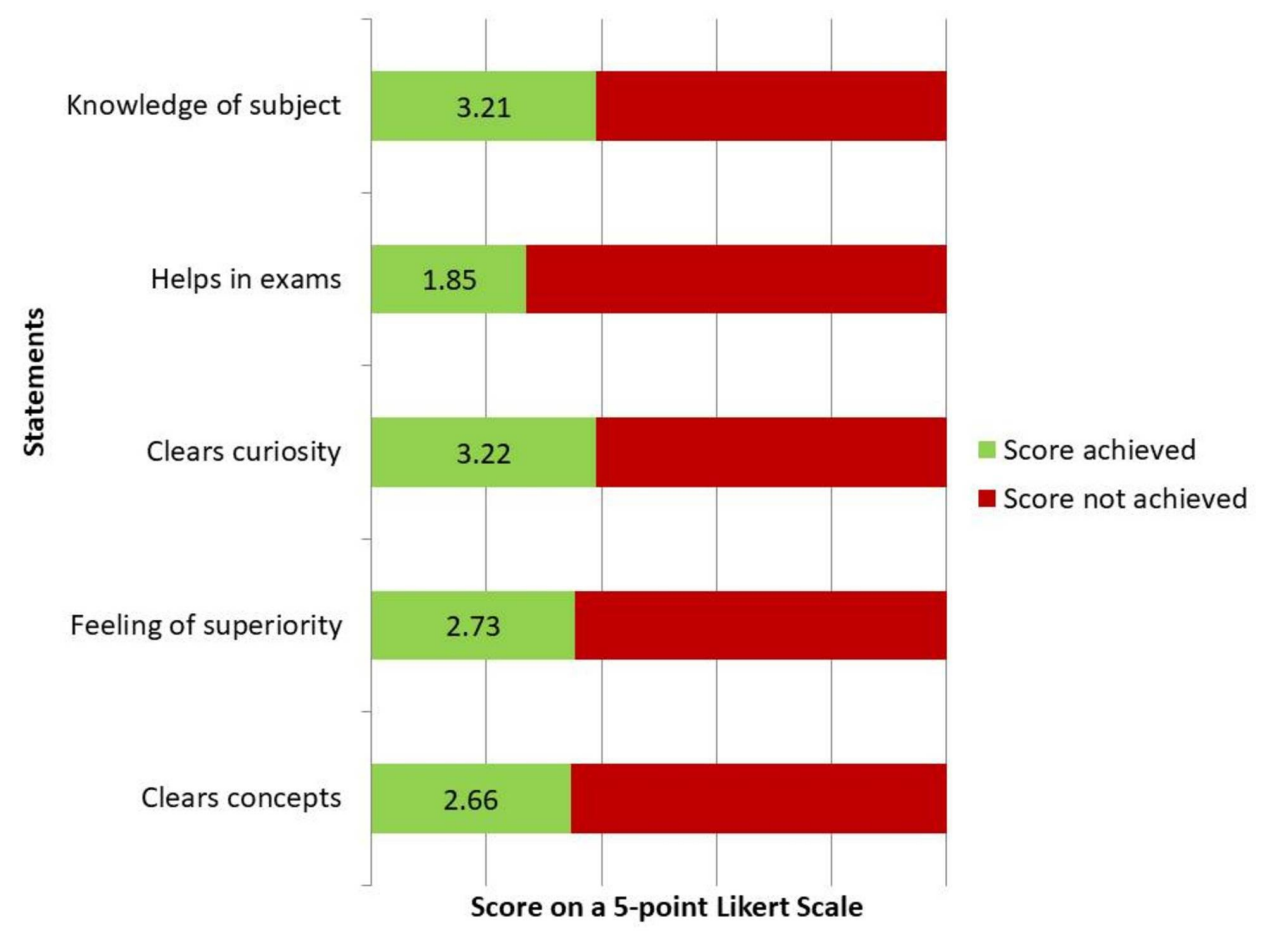
Benefits to the students of using research journals (on a scale of 1 to 5)

Students acknowledged the importance of research journals for an in-depth knowledge of the subject and to clear their concepts. However, the latest research was given a low score with regards to preparation in professional examinations (Figure 2).

**Figure 3:**
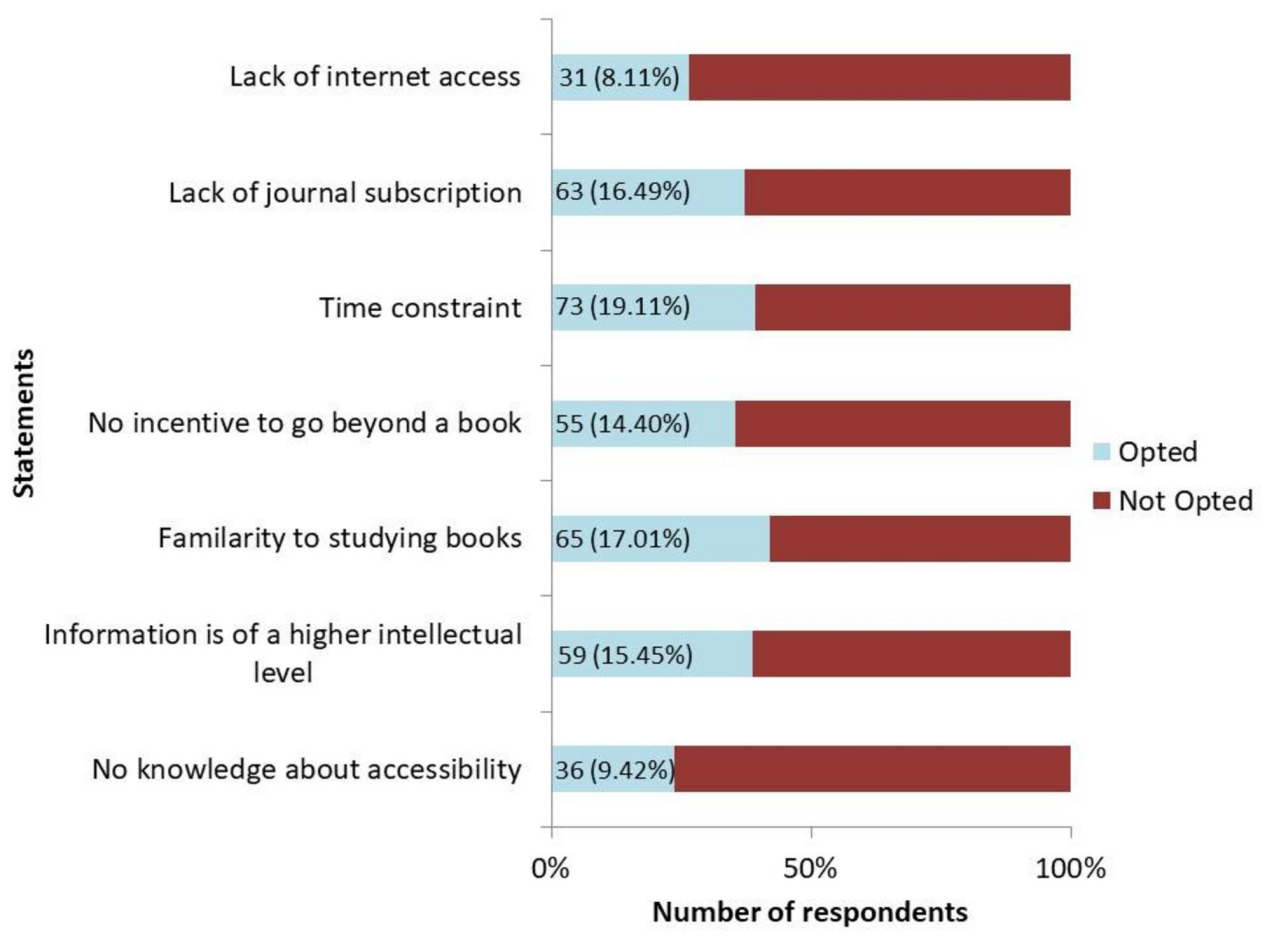
Barriers restricting students from using research journals as a learning tool

Furthermore, 60% students explained the underutilization of journals as a resource material due to the less promptness and motivation of the students to go beyond the books and their lack of familiarity to consult other learning tools besides books. A total of 15% students also revealed that the information in journals is beyond their intellectual level (Figure 3).

## Discussion

The results of our study helped us to probe into various reasons and provided us insight into students’ perception of consulting research journal articles as a study tool. The literature suggests that current medical practices, evidence-based instruction models, and adult-learning theories in medical education have been encouraging the utilization of online medical literature databases and, consequently, the use of research articles, especially e-journals, is getting immense importance as an adjuvant source of learning in an undergraduate medical curriculum [7]. These journals provide the necessary depth that students can use to enhance their learning and become acquainted with the skills required to apply the acquired knowledge in concordance with the principles of evidence-based medicine [8].

According to medical educationists, students should have the optimum skills in applying evidence-based knowledge to patient care, and, for this reason, they must be proficient enough to find relevant and recent research articles and be capable of their critical appraisal [9-10]. In our study, a majority of the students did not use research journals as a study tool even though they did realize that such journals provide good holistic knowledge on relevant topics. Those students who did pursue research journals overwhelmingly used the Internet and did not prefer the institutional library, which elucidates the lack of awareness and training to search online medical literature databases. These aspects need to be explored further and highlight the need for different pedagogical means to train students to access available resources.

Students also appreciated the importance of research journals to solve the problems faced during the learning process and considered it as a means to gain an edge over other students. However, students didn’t perceive research journals as a resource for preparing for their exams. This particular aspect necessitates exploring the factors responsible for this underutilization, as it could imply the lack of incorporation of the latest evidence-based research articles and clinical up-to-date knowledge in routine assessments.

## Conclusions

In our scenario, students, despite being aware of the importance of research articles, continue to use the conventional method of studying. A number of reasons were found for the underutilization of this effective learning tool in the recent era of medical advancements. Over-familiarity with books, less encouragement by the faculty, and lack of training on methods of accessibility to research journals were some of the reasons highlighted by our research study. These issues should be addressed by medical educationists to obtain the maximum use of modern learning strategies. One strategy can be to use such articles as text references for discussions in class. The students can also be assessed on the content of the article that has been discussed. Additionally, the students can also be instructed to present relevant articles in form of a multimedia presentation.

## References

[1] Altman DG (2002). Poor-quality medical research. What can journals do?. JAMA.

[2] Sackett DL, Rosenberg WMC, Gray JAM, Haynes RB, Richardson WS (1996). Evidence-based medicine: what it is and what it isn’t. BMJ.

[3] Taheri H, Mirmohamadsadeghi M, Adibi I, Ashorion V, Sadeghizade A, Adibi P (2008). Evidence-based medicine (EBM) for undergraduate medical students. Ann Acad Med Singapore.

[4] Peterson MW, Rowat J, Kreiter C, Mandel J (2004). Medical students’ use of information resources: is the digital age dawning?. Acad Med.

[5] Jeelani W, Aslam SM, Elahi A (2014). Current trends in undergraduate medical and dental research: a picture from Pakistan. J Ayub Med Coll Abbottabad.

[6] Simon FA, Aschenbrener CA (2005). Undergraduate medical education accreditation as a driver of lifelong learning. J Contin Educ Health Prof.

[7] Nicholas D, Rowlands I, Williams P (2011). E-journals, researchers — and the new librarians. Learn Publ.

[8] Just ML (2012). Is literature search training for medical students and residents effective? A literature review. J Med Libr Assoc.

[9] Cullen R, Clark M, Esson R (2011). Evidence-based information-seeking skills of junior doctors entering the workforce: an evaluation of the impact of information literacy training during pre-clinical years. Health Info Libr J.

[10] Haseeb A, Bilal M, Ansari MA (2016). Impact of mode of curriculum on knowledge and attitudes of medical students towards health research. J Clin Diagn Res.

